# Supplementary material for: esRAGE-expressing oHSV enhances anti-tumor efficacy by inhibition of endothelial cell activation
Source: Mol Ther Oncolytics. 2023 Jan 16;28:171–81. doi: 10.1016/j.omto.2023.01.003 (PMC9918391; doi:10.1016/j.omto.2023.01.003)
Supplement: Document S1. Figures S1–S3 and Tables S1 and S2 [file mmc1.pdf]

OMTO, Volume *28*

Supplemental information

esRAGE-expressing oHSV enhances anti-tumor efficacy by inhibition of endothelial cell activation

Jessica Swanner, Ji Seon Shim, Kimberly A. Rivera-Caraballo, Karina Vázquez-Arreguín, Bangxing Hong, Alberto J. Bueso-Perez, Tae Jin Lee, Yeshavanth Kumar Banasavadi-Siddegowda, Balveen Kaur, and Ji Young Yoo

Supplemental Materials

Figure S1.

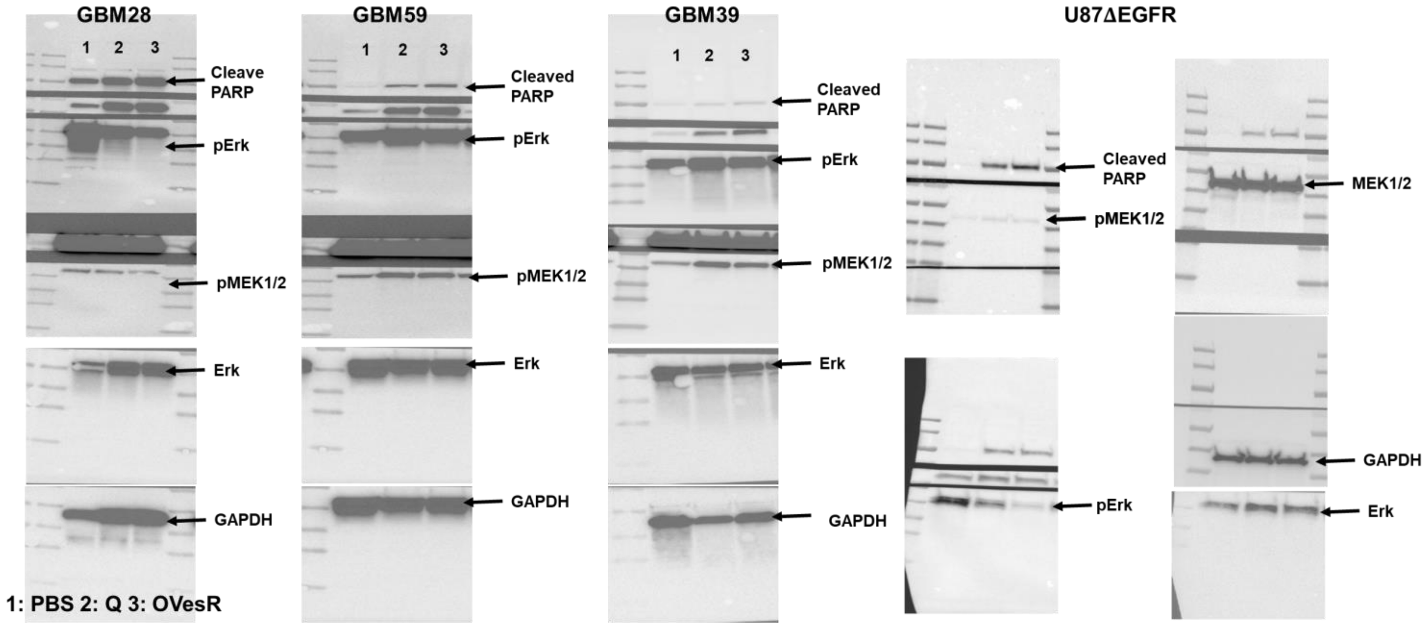

Figure S1. Original full-length image of western blotting analysis used in the Figure 4E. The immunoreactive bands on nitrocellulose membranes were visualized with enhanced chemiluminescence (ECL) (GE Healthcare, Piscataway, NJ, USA) using ChemiDoc MP (Bio-Rad, Hercules, CA, USA). Lane 1: PBS; Lane 2: Q; Lane 3: OVesR.

Figure S2.

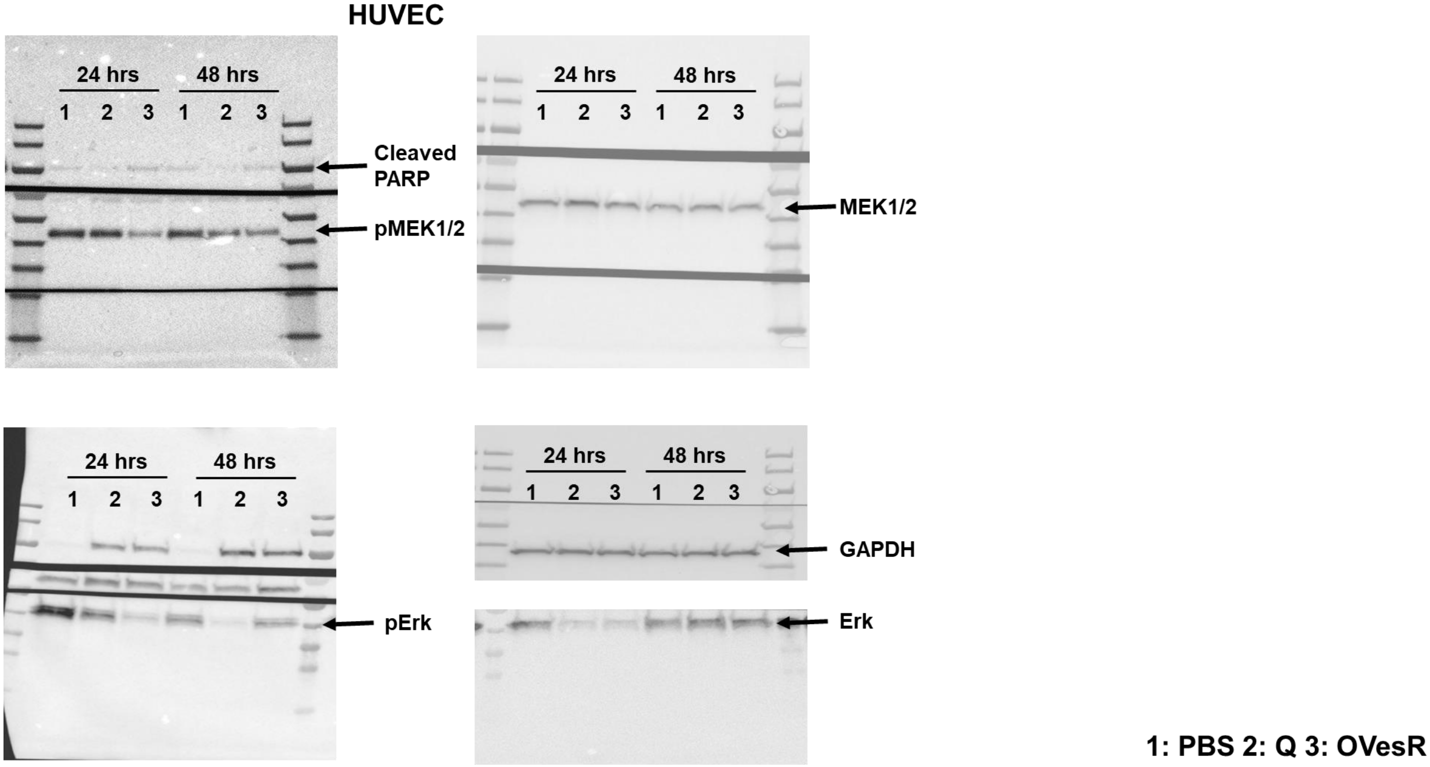

Figure S2. Original full-length image of western blotting analysis used in the Figure 5C. The immunoreactive bands on nitrocellulose membranes were visualized with enhanced chemiluminescence (ECL) (GE Healthcare, Piscataway, NJ, USA) using ChemiDoc MP (Bio-Rad, Hercules, CA, USA). Lane 1: PBS; Lane 2: Q; Lane 3: OVesR.

Figure S3.

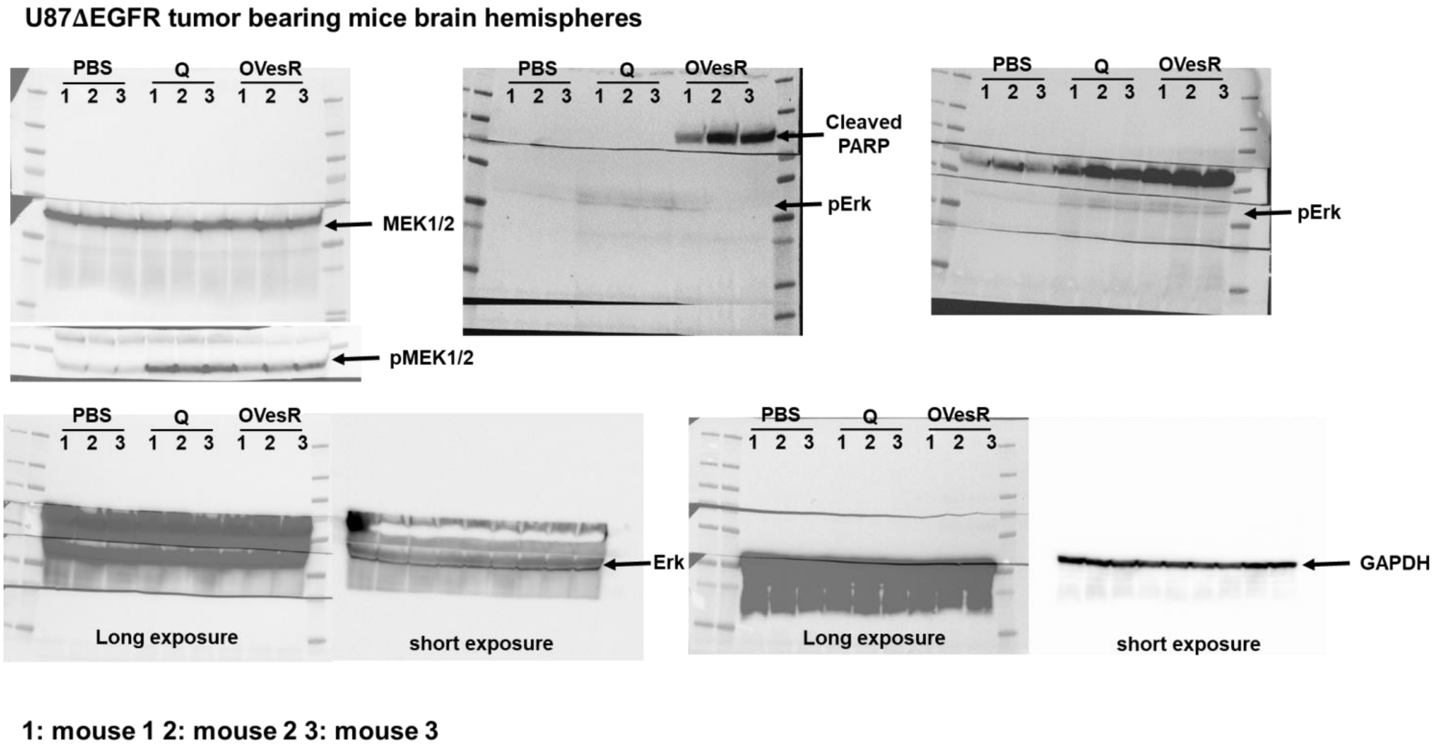

Figure S3. Original full-length image of western blotting analysis used in the Figure 6B. The immunoreactive bands on nitrocellulose membranes were visualized with enhanced chemiluminescence (ECL) (GE Healthcare, Piscataway, NJ, USA) using ChemiDoc MP (Bio-Rad, Hercules, CA, USA). Long and short exposure images were provided for total Erk and GAPDH. Numbers 1, 2, and 3 stand for each mouse.

Table S1. List of the primers used in the study

hCCL5	Forward	5'-CCTGCTGCTTTGCCTACATTGC-3'
	Reverse	5'-ACACACTTGGCGGTTCTTTCGG-3'
hVCAM1	Forward	5'-GTGGAGCTCTACTCATTCCCTA-3'
	Reverse	5'-CTCCAGAATAGTCTCCCCCTTAAG-3'
hICAM1	Forward	5'-GGTAGCAGCCGCAGTCATAA-3'
	Reverse	5'-TCCCTTTTTGGGCCTGTTGT-3'
GAPDH	Forward	5'-CCAGCAAGGACACTGAGCAA-3'
	Reverse	5'-GGGATGGAAATTGTGAGGGA-3'

Table S2. List of the antibodies used in the study

Antibody	Cat#	Vendor	Dilution
Cleaved PARP	9541	Cell signaling Technology	1:1000
p-AKT	9271	Cell signaling Technology	1:1000
p-ERK	4370	Cell signaling Technology	1:1000
T-ERK	9102	Cell signaling Technology	1:1000
T-AKT	4691	Cell signaling Technology	1:1000
p-MEK	9154	Cell signaling Technology	1:1000
GAPDH	2118	Cell signaling Technology	1:2000
T-MEK	9126	Cell signaling Technology	1:1000
Anti-rabbit IgG, HRP-linked Antibody	7074	Cell signaling Technology	1:2000
