# Supplementary material for: esRAGE-expressing oHSV enhances anti-tumor efficacy by inhibition of endothelial cell activation
Source: Mol Ther Oncolytics. 2023 Jan 16;28:171–81. doi: 10.1016/j.omto.2023.01.003 (PMC9918391; doi:10.1016/j.omto.2023.01.003)
Supplement: Document S2. Article plus supplemental information [file mmc2.pdf]

Molecular Therapy
Oncolytics
Original Article

esRAGE-expressing oHSV enhances anti-tumor efficacy by inhibition of endothelial cell activation

Jessica Swanner,[1] Ji Seon Shim,[1] Kimberly A. Rivera-Caraballo,[1,2] Karina Vázquez-Arreguín,[1,2] Bangxing Hong,[1,2] Alberto J. Bueso-Perez,[1] Tae Jin Lee,[1] Yeshavanth Kumar Banasavadi-Siddegowda,[3] Balveen Kaur,[1,2] and Ji Young Yoo[1]

[1]Department of Neurosurgery, McGovern Medical School, University of Texas Health Science Center at Houston, 6431 Fannin St., MSE R117A, Houston, TX 77030, USA; [2]Georgia Cancer Center and the Department of Pathology, Augusta University, 1410 Laney Walker Blvd, CN-3311, Augusta, GA 30912, USA; [3]Surgical Neurology Branch, National Institute of Neurological Disorders and Stroke, National Institutes of Health, Bethesda, MD 20892, USA

High-mobility group box 1 (HMGB1) is a damage-associated molecular pattern (DAMP) molecule that plays an important role in inflammation and tumorigenesis. Receptor for advanced glycation end products (RAGE) is one of the major receptors to which extracellular HMGB1 binds to mediate its activity. RAGE is highly expressed on the endothelial cells (ECs) and regulates endothelial permeability during inflammation. Here, we introduced the endogenous secretory form of RAGE (esRAGE) as a decoy receptor for RAGE ligands into an oncolytic herpes simplex virus 1 (oHSV) (OVesRAGE), which, upon release, can function to block RAGE signaling. OVesRAGE significantly decreased phosphorylation of MEK1/2 and Erk and increased cleaved PARP in glioblastoma (GBM) cells *in vitro* and *in vivo*. oHSV-infected GBM cells co-cultured with ECs were used to test OVesRAGE effect on EC activation, vessel leakiness, virus replication, and tumor cell killing. OVesRAGE could effectively secrete esRAGE and rescue virus-induced EC migration and activation. Reduced EC activation facilitated virus replication in tumor cells when co-cultured with ECs. Finally, OVesRAGE significantly enhanced therapeutic efficacy in GBM-bearing mice. Collectively, our data demonstrate that HMGB1-RAGE signaling could be a promising target and that its inhibition is a feasible approach to improve the efficacy of oHSV therapy.

INTRODUCTION

Glioblastoma (GBM) is the most aggressive primary brain malignancy in adults, and the median survival time is about 18 months.[1,2] Nearly all tumors recur and are often resistant to chemotherapy, leaving few options available to delay disease progression.[3] Oncolytic herpes simplex virus 1 (oHSV) therapy is the most advanced FDA-approved virotherapy for patients with advanced unresectable melanoma and has recently gained conditional approval in Japan for treatment of GBM.[4,5] oHSV therapy destroys tumor cells by direct lytic destruction and also instigates the activation of anti-tumor immune response. However, the ultimate success of this anti-tumor response is restrained by a suppressive inflammatory tumor microenvironment (TME) often aggravated by oHSV treatment.[6]

We and others have previously identified that oHSV infection triggers the release of a high-mobility group box 1 (HMGB1) secretion into the TME, and this results in increased tumor edema that can limit oHSV efficacy.[7–13] Among several receptors that convey HMGB1-induced signaling, the receptor for advanced glycation end products (RAGE) is increased in many cancers, including those derived from the breast, colon, pancreas, and brain, and has been associated with autophagy, proliferation, angiogenesis, and resistance to chemotherapy.[14–18] Since it is constitutively expressed during embryonic development and expressed at low levels in normal tissues after birth, it makes a good druggable target. RAGE activation promotes an inflammatory and pro-tumorigenic environment that can suppress anti-tumor immunity. Several studies have demonstrated that inhibition of RAGE signaling increases survival in a number of cancer models,[18,19] with some reports of a multiple spliced and secreted variant of endogenous RAGE (esRAGE) whose levels correlated with decreased cancer risk and increased overall survival.[20]

Here, we evaluated the impact of blocking RAGE signaling on oHSV therapy. We generated a novel therapeutic oHSV (OVesRAGE) that secretes esRAGE and neutralizes RAGE signaling by competing with full-length RAGE for ligands. We found that esRAGE expression in conjunction with oHSV therapy improves oHSV efficacy by reducing endothelial cell (EC) activation and recruitment into the

Received 23 August 2022; accepted 12 January 2023;
https://doi.org/10.1016/j.omto.2023.01.003.

Correspondence: Balveen Kaur, PhD, Georgia Cancer Center and the Department of Pathology, Augusta University, 1410 Laney Walker Blvd, CN-3311, Augusta, GA 30912, USA.
E-mail: bkaur@augusta.edu
Correspondence: Ji Young Yoo, PhD, Department of Neurosurgery, University of Texas Health Science Center at Houston, 6431 Fannin St., MSE R117A, Houston, TX 77030, USA.
E-mail: ji.young.yoo@uth.tmc.edu

This is an open access article under the CC BY license (http://creativecommons.org/licenses/by/4.0/).

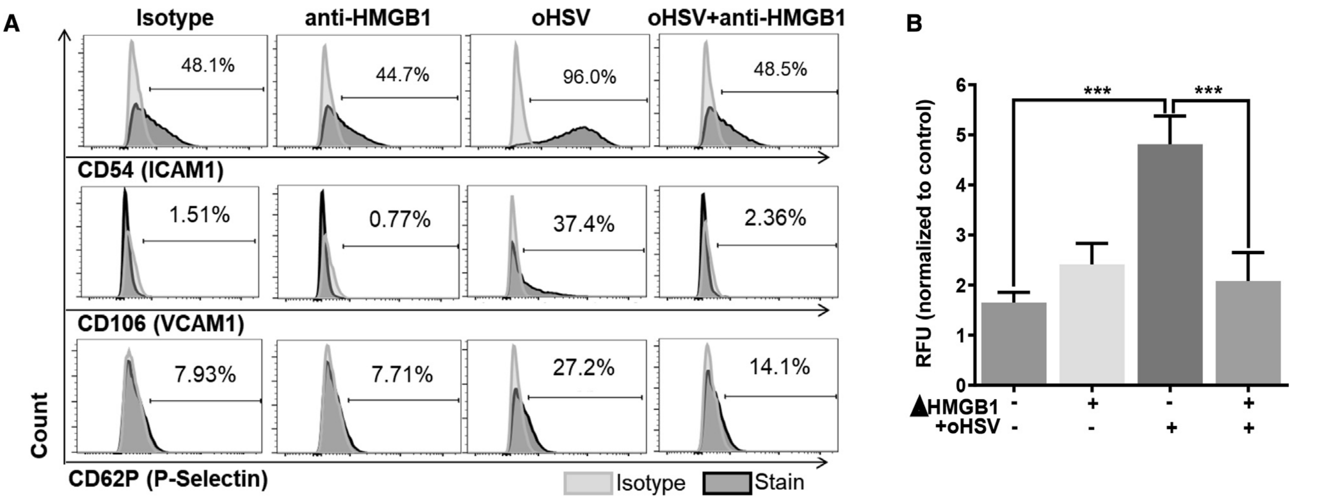

Figure 1. oHSV therapy-induced HMGB1 activates endothelial cells, hindering therapeutic efficacy of oHSV *in vitro*

(A) Endothelial cells (ECs) were stimulated with conditioned media (CMs) collected from oHSV infected/uninfected LN229 cells ± αHMGB1, and changes in receptor expression were assessed by flow cytometry (*p ≤ 0.05; **p ≤ 0.01).

(B) HMGB1 depletion reduces EC activation upon oHSV infection, as indicated by peripheral blood mononuclear cell (PBMC) binding. ECs were stimulated with CM collected from oHSV infected/uninfected LN229 cells ± αHMGB1. Fluorescently labeled PBMCs were then overlaid, and number of bound PBMC were quantified by counting. (***, p≤0.001). Data shown is quantification of PBMC cells bound to EC.

brain TME. Furthermore, we show that OVesRAGE increases viral replication efficacy by allowing enhanced viral penetrance, reducing membrane-bound RAGE signaling through sequestration of available ligands and through modulation of the inflammatory immune response to oncolytic virus (OV) infection. The knowledge gained through this study will help guide future OV research and will contribute to the scientific understanding of RAGE signaling within the TME.

RESULTS

oHSV therapy-mediated HMGB1 release acts as paracrine stimulator for EC activation

We have previously shown that HMGB1 secreted from tumor cells infected with oHSV increases vascular leakage and edema, but administration of an HMGB1-blocking antibody inhibits this effect.[7] In this study, we used rHSVQ that was doubly deleted for the neuro-virulence factor γ-34.5 and has a disrupted ICP6 and evaluated the effects of HMGB1 blockade on EC function. Utilizing flow cytometry, ECs stimulated with conditioned media (CMs) from uninfected or oHSV (rHSVQ)-infected glioma cells in the presence/absence of neutralizing HMGB1 antibody (oHSV-infected/uninfected CM ± αHMGB1) were assessed for changes in expression of cell surface markers, indicative of EC activation. Treatment of human umbilical vein ECs (HUVECs) with CM from oHSV-infected glioma cells increased expression of EC activation surface receptors: CD54 (ICAM1), CD106 (VCAM1), and CD62P (P-selectin) (Figure 1A). Elevated receptor expression was reduced to near-control levels in the presence of an HMGB1-blocking antibody. In order to test if HMGB1 also directly modulated EC function, we tested the ability of CMs derived from infected glioma cells ± HMGB1 neutralizing antibody to activate ECs. Figure 1B shows reduced EC activation (as measured by peripheral blood mononuclear cell [PBMC] binding) in the presence of infected glioma cell CM with an HMGB1 neutralizing antibody. Together, these results indicate that oHSV therapy-triggered secreted HMGB1 activates ECs.

RAGE blockade inhibits oHSV therapy-induced EC migration and leakiness

HMGB1 signals through the RAGE as well as the Toll-like receptors (TLRs), especially TLR4. RAGE is a multi-ligand receptor that binds AGEs, and these occur largely in response to reactive oxygen species (ROS), HMGB1, s100 proteins, and amyloid β. To evaluate gene expression changes in oHSV-infected cells that significantly affected pathways involved in HMGB1 signaling, we analyzed RNA sequencing results from oHSV (rHSVQ)-infected U87ΔEGFR glioma cells using a Kyoto Encyclopedia of Genes and Genomes (KEGG) enrichment analysis.[21,22] We observed a significant enrichment of RAGE and Rap signaling pathways, which are downstream of HMGB1 signaling following infection (Figure 2A). RAGE is highly expressed in tumor-associated macrophages, and ECs have also been linked to glioma-associated inflammation and angiogenesis.[17,18] Thus, we tested the impact of RAGE receptor on oHSV-induced EC activation using a function-blocking antibody and endogenous secretory RAGE (esRAGE) peptide. To measure the impact of HMGB1-RAGE signaling on vascular permeability, HUVEC monolayers grown on 8 μm Transwell chambers were stimulated with CMs from uninfected or oHSV-infected glioma cells in the presence or absence of a RAGE-blocking antibody (2 μg/mL) or purified esRAGE (200 ng/mL), and the amount of Evan's Blue albumin (EBA) that permeated the monolayer to the lower chamber was measured to

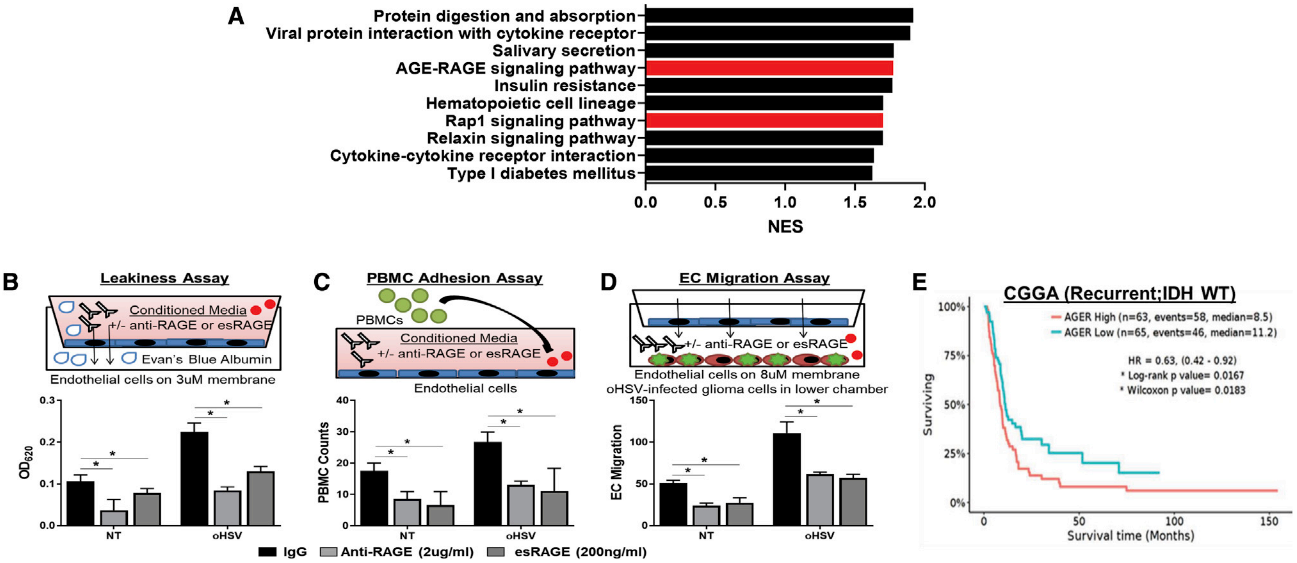

Figure 2. HMGB-1 RAGE signaling induced by oHSV therapy activates endothelial cells

(A) Total RNA sequencing of U87ΔEGFR cells analyzed for top genes involved in inflammatory pathways.[21] KEGG pathway analysis showing the top 10 upregulated pathways involved in inflammatory regulation enriched in oHSV-infected U87ΔEGFR cells cultured with microglial cells.

(B) EC leakiness was evaluated by measuring the ability of Evan's Blue albumin (EBA) to permeate a confluent EC monolayer as shown on the top. Data shown are mean EBA ± SD.

(C) PBMC adhesion to EC monolayers was assessed as shown on the top. Data shown are mean PBMC ±SD bound to ECs stimulated with CM ± oHSV ± αHMGB1.

(D) Quantification of EC migration after ECs were stimulated by glioma cells ± oHSV ± anti-RAGE (2 μg/mL) or esRAGE (200 ng/mL). Data shown are the mean numbers of ECs that migrated through the Transwell membrane ± SD. *$p < 0.05$.

(E) Kaplan-Meier survival curve are plotted using patients with IDH wild-type recurrent glioma with overall survival information from the Chinese Glioma Genome Atlas (CGGA). Tumors are stratified into two groups with the cutoff set as the median of AGER gene expression. Log rank and Wilcoxon p values are labeled on top of each survival curve. *$p < 0.05$. Data shown are RFUs ± SD, normalized to media control.

evaluate vascular leakiness (Figure 2B). Treatment of ECs with CMs from oHSV-infected glioma cells resulted in a significant increase in permeability that was rescued in the presence of a RAGE-blocking antibody and purified esRAGE (Figure 2B). Next, since endothelial activation also increases adhesion of leukocytes, we quantified the adhesion of human donor-derived PBMCs to ECs treated with CMs from oHSV-infected and uninfected tumor cells. Consistent with the increased EC activation, we observed a significant increase in PBMC adhesion to ECs treated with CMs from oHSV-infected glioma cells that was rescued in the presence of RAGE antibody (2 μg/mL) and purified esRAGE (200 ng/mL) (Figure 2C). Furthermore, we used a Boyden chamber assay to measure changes in EC migration (Figure 2D). Stimulating ECs with CMs from oHSV (rHSVQ)-infected glioma cells resulted in a significant increase in EC migration, which was rescued in the presence of a RAGE-blocking antibody (2 μg/mL) and purified esRAGE (200 ng/mL) (Figure 2D). Together, these results indicate that blocking RAGE signaling significantly inhibits oHSV therapy-induced EC activation and migration. Based upon these data, we hypothesize that RAGE ligands (such as HMGB1) released from oHSV-infected GBM cells that undergo oncolysis activate RAGE signaling within the TME, hindering efficient virus propagation. RAGE expression is increased in many cancers, including those derived from the breast, colon, pancreas, and brain, and has been associated with autophagy, proliferation, angiogenesis, and resistance to chemotherapy.[14–18] To validate the clinical relevance of RAGE expression in patients with GBM, we interrogated data from the Chinese Glioma Genome Atlas (CGGA) accessed via GlioVis.[23] The increased expression of RAGE negatively correlated with the survival of patients with recurrent IDH wild-type GBM (Figure 2E).

Generation of esRAGE-expressing novel therapeutic oHSV, OVesRAGE

esRAGE is an alternatively spliced variant of the full-length RAGE, which lacks the transmembrane domain and cytosolic tail required for signal transduction. This variant acts as a decoy receptor, binding and sequestering RAGE ligands and thus interfering with their RAGE-mediated signal transduction.[3,4] To investigate the impact of blocking this pathway in conjunction with oHSV therapy on efficacy, we generated a novel oHSV that expresses esRAGE within the HSV-1 F-strain backbone, which has been doubly deleted for the neuro-virulence factor γ-34.5 and has a disrupted ICP6 (Figure 3A).[24] To evaluate esRAGE expression and secretion, three primary GBM cells were infected with rHSVQ or OVesRAGE, CM was collected 24 h later, and the expression and secretion of esRAGE were evaluated by western blot analysis (Figure 3B). The secretion of esRAGE was further

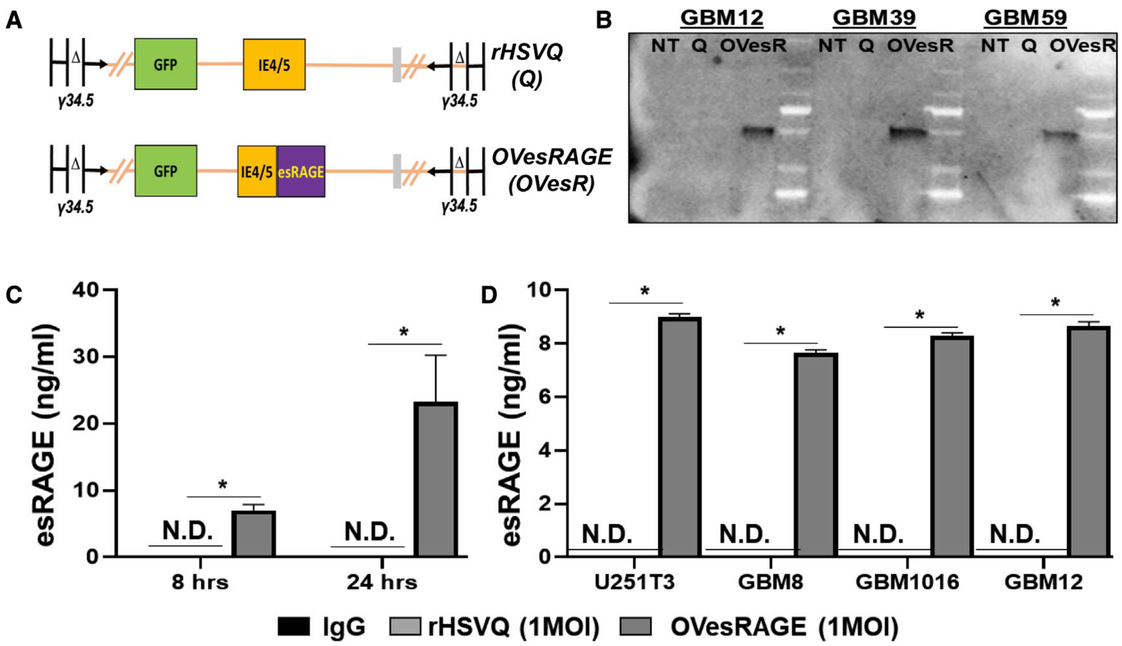

Figure 3. Generation of esRAGE-expressing oHSV (OVesRAGE)

(A) Graphical representation of the F-strain HSV backbone and modified loci: F-strain HSV backbone showing doubly deleted γ34.5 genes and an insertional ICP6 mutation. Expanded ICP6 loci of rHSVQ and OVesRAGE are shown. rHSVQ contains an insertional EGFP, and OVesRAGE contains the esRAGE transgene, which is driven by the IE4/5 promotor. The cartoon shows a structure of control rHSVQ and OVesRAGE.

(B) OVesRAGE virus expresses and secretes esRAGE. Patient-derived primary GBM cells were infected with 0.2 MOI rHSVQ and OVesRAGE virus. Twenty-four h post virus infection, CM was collected, and secretion of esRAGE was confirmed by western blot analysis. Q stands for rHSVQ, and OVesR stands for OVesRAGE.

(C and D) Quantification of secreted esRAGE by OVesRAGE infection of GBM cells.

(C) CMs were collected from 1 MOI rHSVQ- or OVesRAGE-infected U251T3 cells at 8 and 16 h post infection.

(D) A GBM cell line (U251T3) and 3 primary GBM cells (GMB8, GBM1016, and GBM12) were infected with 1 MOI rHSVQ or OVesRAGE, and esRAGE was quantified from the CM 24 h later using an ELISA for esRAGE. Detectable levels of esRAGE were observed in the OVesRAGE-infected cells. *$p < 0.05$.

validated by utilizing an esRAGE-specific ELISA (Figures 3C and 3D).[25] U251T3 GBM cells were infected with rHSVQ or OVesRAGE (MOI = 1), and CM was collected 8 and 24 h later. ELISA data show that esRAGE secretion increased temporally (Figure 3C) and in all OVesRAGE-infected glioma cells (Figure 3D). No detectable esRAGE was observed in the uninfected control or rHSVQ-infected GBM cells, confirming that esRAGE is secreted from OVesRAGE-infected GBM cells (Figures 3C and 3D).

OVesRAGE virus replicates efficiently *in vitro*

Next, we compared the impact of esRAGE expression on viral replication in GBM cells by measuring GFP-positive infected cells using the Cytation 5 live plate reader/imager (Figure 4A). While OVesRAGE appeared to have slightly superior kinetics of virus replication in GBM12, GBM39, and GBM59, there is no significant difference in GBM6 and GBM12 (Figure 4A). Next, we compared the effect of esRAGE expression on virus replication (Figures 4B and 4C). Figure 4B shows the representative immunofluorescent images of mCherry-positive GBM cells infected with control rHSVQ and OVesRAGE. There was no obvious difference in GFP-positive infected cells, and titration of infected cells also revealed that there was no significant difference in virus titer between OVesRAGE and rHSVQ on GBM cells (Figure 4C). To test the effect of esRAGE expression on oHSV therapy-mediated GBM cell killing, we compared the *in vitro* cytotoxicity of several patient-derived primary GBM cells infected with OVesRAGE with that of rHSVQ. Patient-derived primary GBM cells were infected with various MOIs of control rHSVQ or OVesRAGE. Seventy-two h post virus infection, cell viability was measured by MTT assay (Figure 4D). In general, the expression of esRAGE does not negatively affect the cytotoxic potential of oHSV. Western blot data show that OVesRAGE significantly inhibits downstream of RAGE signaling as measured by decreased pErk and pMEK and increased cleaved PARP (Figure 4E). Collectively, these results indicate that esRAGE expression by an oHSV did not negatively affect the ability of oncolytic HSV to infect, replicate, or kill GBM cells *in vitro*.

OVesRAGE moderates EC activation *in vitro*

To evaluate the effect of OVesRAGE on ECs, we measured the migration of ECs toward CM from rHSVQ- or OVesRAGE-infected U251T3 glioma cells used as a chemoattractant in the bottom chamber. The migrated cells on the bottom side of the Transwell were

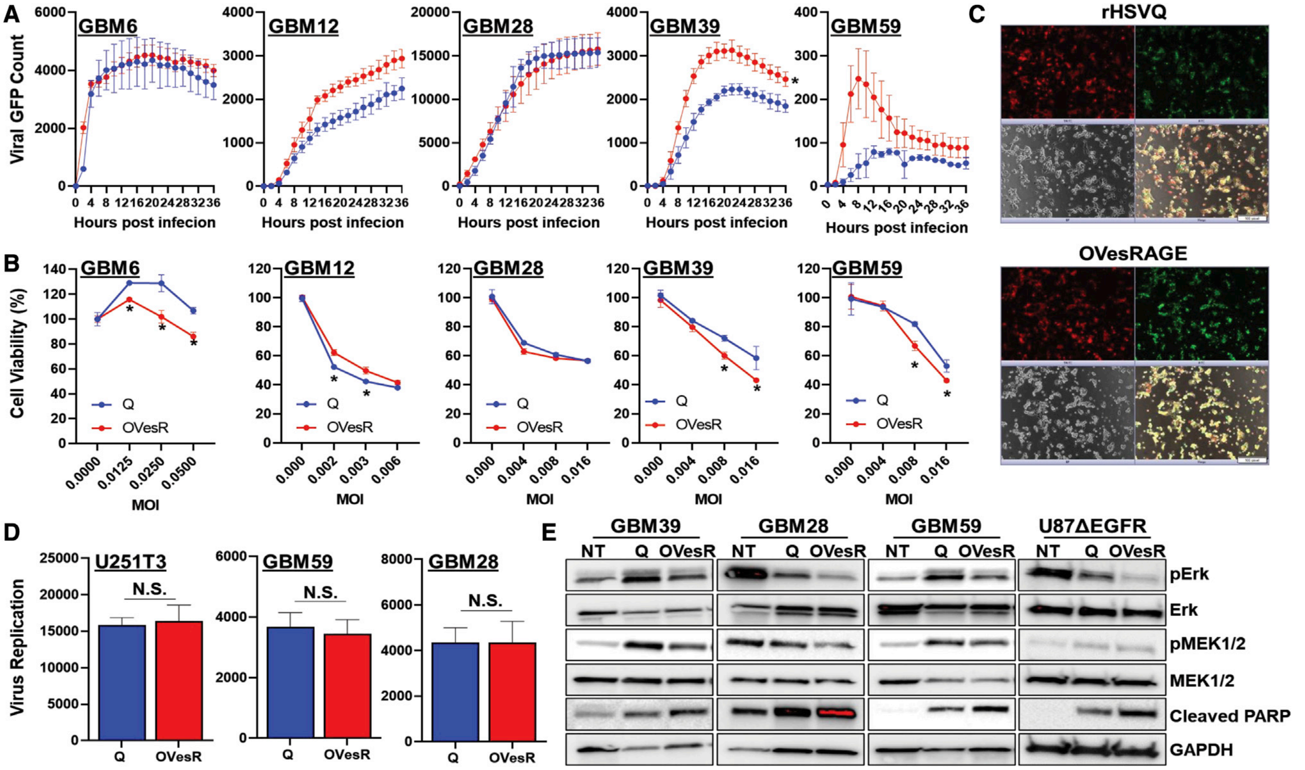

Figure 4. Characterization of OVesRAGE replication and cytotoxicity toward glioma cells

(A) Primary GBM cells were plated on 24-well plates and treated with rHSVQ and OVesRAGE virus for 1 h. Unbound virus was removed, and plate was imaged for 48 h using the Cytation 5 live plate reader/imager. Viral propagation was measured via virus-encoded GFP expression (n = 4/g).

(B) Primary GBM cells were infected with various MOIs of rHSVQ or OVesRAGE, and cell viability was measured by MTT assay at 72 h post virus infection. Data shown are percentage of viable cells relative to uninfected controls ± SD (n = 4/g).

(C and D) U251T3-mCherry cells were infected with 0.05 MOI of rHSVQ or OVesRAGE. Forty-eight h post virus infection, cells and media were harvested, and virus titer was quantified using standard plaque-forming assay.

(C) Representative fluorescence microscopy images of GFP-positive oHSV-infected GBM cells.

(D) Comparison of HSVQ and OVesRAGE viral replication and propagation. Data shown are median titers from cultures ± SD (n = 3/group).

(E) Western blot analysis of the indicated patient-derived primary GBM and U87ΔEGFR glioma cells treated with 0.01–0.05 MOI rHSVQ and OVesRAGE. Twenty-four h post treatment, cells were harvested, and cell lysates were probed with antibodies against pERK, pMEK1/2, total ERK, total MEK1/2, and cleaved PARP. Glyceraldehyde 3-phosphate dehydrogenase was used as a loading control.

visualized and quantified. Figure 5A shows a statistically significant reduction in the migration of ECs toward CM derived from OVesRAGE-infected glioma cells compared with rHSVQ-treated CMs (p = 0.0004). Next, we measured the gene expression of ICAM, VCAM, and CCL5 (EC activation markers) on ECs after infection. Briefly, U251T3 cells were seeded on the plate and infected with phosphate-buffered saline (PBS), rHSVQ, or OVesRAGE (MOI = 0.2). One h post virus infection, unbound virus was removed, CM was replaced with media containing 0.4% human immunoglobulin G (IgG), and serum-starved ECs were placed on top of a 0.3-μM Boyden chamber. Twenty-four h after the co-culture, ECs from the upper chamber were harvested, and the gene expression for EC activation (ICAM, VCAM, and CCL5) was evaluated using quantitative real-time PCR (Figure 5B). Consistent with our previous observations,[22] there is significantly increased ICAM1, VCAM1, and CCL5 gene expression in control rHSVQ-infected cells. However, there was significant reduction of ICAM1, VCAM1, and CCL5 in the ECs co-cultured with OVesRAGE-infected CMs compared with control rHSVQ (Figure 5B). Western blot data show that OVesRAGE treatment significantly inhibited pMEK and pErk and increased cleaved PARP in ECs (Figure 5C). To evaluate the effect of EC activation on virus replication with and without esRAGE, we investigated virus replication, propagation, and tumor cell killing in glioma-EC co-cultures. Stably mCherry-expressing U251T3 cells (U251T3-mCherry) were infected for 1 h with 0.05 MOI rHSVQ or OVesRAGE and then overlaid with ECs. Figure 5D shows a significant increase in GFP-positive virus-infected cells in OVesRAGE-infected GBM cells overlaid with ECs compared with the rHSVQ-infected tumor cells overlaid with ECs, indicating that OVesRAGE-mediated decreased EC activation resulted in increased

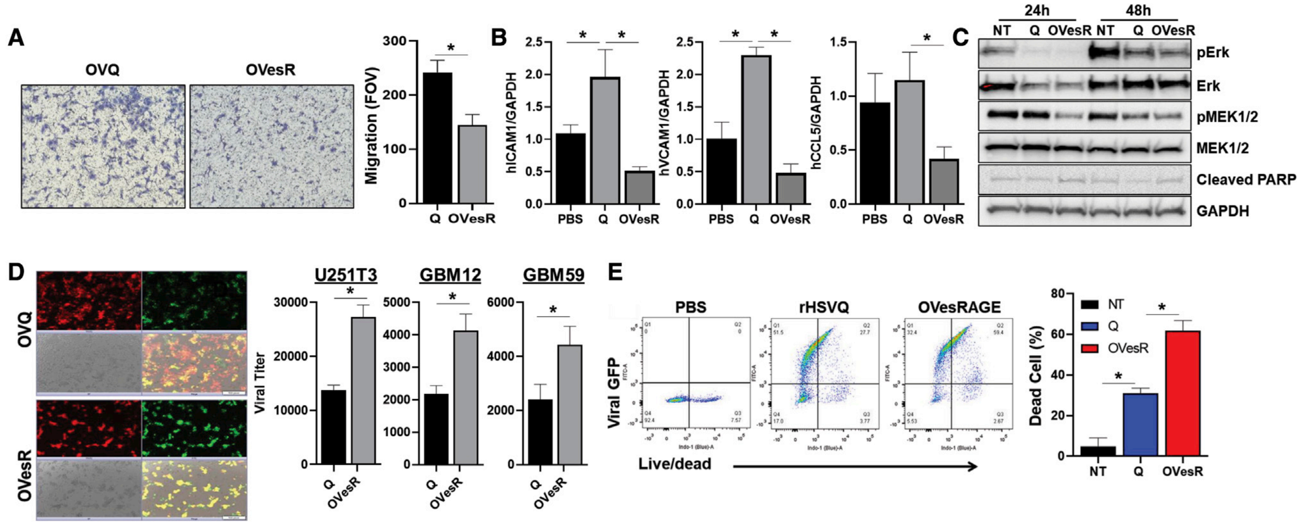

Figure 5. The impact of OVesRAGE on EC function

(A) OVesRAGE reduces EC migration. CMs were collected from U251T3 cells infected with 1 MOI rHSVQ or OVesRAGE for 14 h. CMs were added in the bottom chamber of the plate, and serum-starved ECs were plated on the top chamber and were allowed to migrate. Fourteen h later, cells were fixed and stained with crystal violet. Representative microscopy images of migrated ECs and quantification of EC migration. Data shown are mean number of ECs that migrated through the Transwell membrane ± SD.

(B) OVesRAGE decreases ICAM1, VCAM1, and CCL5 gene expression on ECs. CMs harvested from U251T3 cells infected with rHSVQ or OVesRAGE for 14 h were plated on the bottom plate, and ECs were plated on the 0.3 μM of Transwell on the top and cultured. Twenty-four h later, RNAs were isolated from ECs, quantitative real-time PCR analysis was conducted. Data shown are fold changes in gene expression ± SD relative to GAPDH. *$p < 0.05$.

(C) Significant reduction of pErk and pMEK1/2 in HUVECs treated with OVesRAGE for 24 and 48 h. Glyceraldehyde 3-phosphate dehydrogenase was used as a loading control.

(D) U251T3-mCherry cells were infected with rHSVQ or OVesRAGE at MOI 0.01 and overlaid with ECs. Representative fluorescent microscopy images.

(E) Quantification of virus replication. Various glioma cells (U251T3, GBM12, and GBM59) were infected with 0.01 MOI rHSVQ or OVesRAGE. One h post virus infection, unbound virus was removed and overlaid with ECs and culture for 48 h. Viral titers were quantified using a PFU assay. *$p < 0.05$.

(F) Glioma cell killing (live/dead cell staining) in glioma and EC co-culture was assessed by flow cytometry 48 h post co-culture. *$p < 0.05$.

viral replication and propagation. Consistent with fluorescent microscopy of GFP-positive infected cells (Figure 5D), quantification of virus replication also revealed a significant increase in virus replication in 3 different GBM cells infected with OVesRAGE when co-cultured with ECs compared with rHSVQ-infected GBM cells co-cultured with ECs (U251T3 [1.98-fold, $p < 0.05$], GBM12 [1.9-fold, $p < 0.05$], or GBM59 (1.84-fold, $p < 0.05$]) (Figure 5E). Flow cytometric analysis of live/dead cell staining revealed that while OVesRAGE infection increased oHSV-infected GFP-positive cells (GFP-positive cells, rHSVQ vs. OVesRAGE: 27.7% vs. 59.4%, $p < 0.05$), a significant increase in the percentage of total dead cells was observed in OVesRAGE-infected GBM cells co-cultured with ECs (Figure 5F). Collectively, these data indicate that oHSV infection of GBM cells activates ECs that can inhibit virus replication. OVesRAGE virus effectively inhibited the oHSV-infected GBM cells and EC cross talk, permitting more efficient virus replication and tumor cell killing.

OVesRAGE increases anti-tumor efficacy and virus propagation *in vivo*

To evaluate the therapeutic efficacy of OVesRAGE *in vivo*, 6- to 8-week-old Balb/c nu/nu (GBM30, n = 20 mice/group) or NSG (U87ΔEGFR, n = 8 mice/group) mice were inoculated via stereotactic intracranial implantation (Figure 6A). Seven days post tumor implantation, mice were treated intratumorally with PBS, rHSVQ, or OVesRAGE at a dose of 2×10^5 PFU per mouse. Mice treated with OVesRAGE demonstrated a significant survival advantage compared with control rHSVQ in both intracranial xenografts (U87ΔEGFR: median survival: 26.5 days, $p = 0.0171$ vs. rHSVQ; GBM30: median survival: 28 days, $p = 0.0224$ vs. rHSVQ alone). Western blot analysis of the intracranial U87ΔEGFR tumor-bearing mice brain tissues revealed significant down-regulation of the RAGE signaling pathway in OVesRAGE-treated tumors compared with control rHSVQ-treated tumors, as evidenced by decreased pMEK1/2 and pErk and increased cleaved PARP (Figure 6B). Additionally, tumor-bearing brain hemispheres were harvested 48 h post virus injection from intracranial U87ΔEGFR tumor-bearing mice treated with rHSVQ or OVesRAGE, and viral replication and spread *in vivo* was quantified. Similar to our *in vitro* results, there was a significant increase of virus replication and propagation in the OVesRAGE-treated tumors compared with control rHSVQ virus (Figure 6C). Taken together, these data indicate that OVesRAGE virus inhibits oHSV therapy-induced HMGB1-RAGE signaling in the TME, thereby allowing increased virus spread and enhancing anti-tumor efficacy *in vivo*.

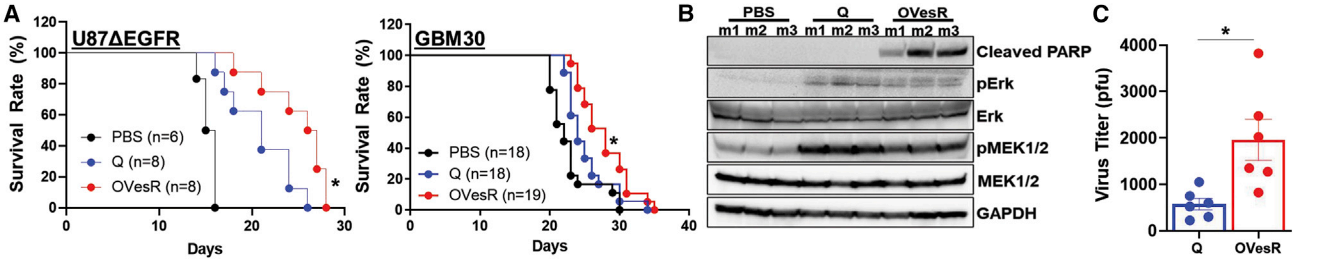

Figure 6. Therapeutic efficacy of OVesRAGE in intracranial glioma-bearing mice

(A) Survival rate of U87ΔEGFR and GBM30 glioma-bearing NSG mice treated with control PBS (black circle), rHSVQ (blue circle), or OVesRAGE (red circle) 7 days post tumor cell implantation. Mice survival data were assessed by log rank (Mantel-Cox) test (*$p < 0.05$).

(B) Western Blot data of U87ΔEGFR tumor-bearing mice brain tissue sections collected 2 days post virus treatment (n = 3/group).

(C) Quantification of virus replication *in vivo*. U87ΔEGFR glioma-bearing NSG mice were injected with 5×10^5 PFU of rHSVQ or OVesRAGE 10 days post tumor implantation. Tumor-bearing brain hemispheres were harvested 2 days post virus injection and processed for the titration as previously described. Virus titers were measured by standard PFU assay on Vero cells (n = 3/group). *$p < 0.05$.

DISCUSSION

oHSV therapy has emerged as a promising biological therapy to treat some of the most aggressive cancers.[26] Results from several ongoing clinical trials in patients with GBM have underscored the promise of this agent for brain tumors. More recently, Delytact (an oHSV drug) was granted conditional approval for the treatment of patients with GBM in Japan. While oHSV therapy offers many benefits over standard radio- and chemotherapy, the efficacy is often diminished for reasons attributed to the TME.[6,7,27,28] Oncolytic viral therapy relies on tumor cell destruction, which has been shown by multiple labs to result in the release of damage-associated molecular patterns (DAMPs) such as ATP, calreticulin, and HMGB1. HMGB1 is a highly conserved protein with multiple functions both within and outside the cell. Numerous reports have previously shown that oHSV treatment of tumor cells results in the release of HMGB1 in the extracellular environment.[7–13] While most of these studies have reported the release of HMGB1 after virotherapy, the impact of HMGB1 appears to depend on its localization. For example, genetic depletion of HMGB1 (which depletes both intra- and extracellular HMGB1) has revealed increased virus replication *in vitro*.[8] On the other hand, overexpression of HMGB1 by an oHSV was shown to mildly increase the cytotoxicity of oHSV in hypoxic colon cancer cells *in vitro*.[29] To specifically delineate the impact of secreted HMGB1 on virus replication, we utilized an HMGB1-blocking antibody. Although blockade of secreted HMGB1 did not affect virus replication *in vitro*, we observed that mice treated with an HMGB1-blocking antibody lived longer, primarily due to reduced edema *in vivo*.[7]

The RAGE receptor is one of the major receptors through which HMGB1 transduces its effect. Here, we show that virus-induced EC activation by HMGB1 is mediated through RAGE. RAGE expression is increased in ECs and immune cells following injury or inflammation and has been associated with a number of inflammatory diseases such as diabetes, stroke, and arthritis. RAGE is a multi-ligand receptor that binds AGEs, which occur largely in response to ROS, HMGB1, several of the s100 proteins, and amyloid β. Ligand interaction with RAGE activates signaling through the myeloid differentiation primary-response protein 88 (MyD88), leading to activation of nuclear factor κB (NF-κB) and secretion of pro-inflammatory cytokines, as well as a positive feedback loop that upregulates RAGE expression.

RAGE expression has also been linked to glioma-associated inflammation and angiogenesis, and its blockade can reduce tumor growth in preclinical studies[17,18,30] Consistent with these studies, expression of esRAGE in patients is associated with decreased cancer risk as well as enhanced overall survival and prognosis.[19,20] Here, we developed an oHSV (OVesRAGE) that secretes esRAGE following tumor cell infection and tested this on various glioma cells *in vitro* and *in vivo*. Our data demonstrate that the modulation of RAGE signaling by OVesRAGE inhibits EC activation, thereby improving anti-tumor efficacy of oHSV.

RAGE has also been postulated to represent a new link between the innate and adaptive immune system. Studies by the Badie group and others have highlighted the importance of RAGE signaling, specifically in macrophages and microglia.[18,25,31,32] Its expression is increased in microglia upon inflammation or injury,[33,34] and it is documented to play a central role in monocyte activation driving the rapid release of interleukin-1β and tumor necrosis factor alpha (TNF-α).[33,35] Combined inhibition of RAGE and HMGB1 was found to have a stronger anti-tumor effect than either RAGE or HMGB1 inhibition alone, suggesting that both RAGE and HMGB1 can affect tumorigenesis independent of each other.[36]

oHSV has been previously shown to alter the TME, resulting in changes in EC functions such as proliferation, migration, and other pro-tumorigenic roles.[37–39] Here, we have shown that modulation of HMGB1-RAGE signaling utilizing esRAGE could improve therapeutic efficacy of oHSV. To our knowledge, this is the first time an oHSV has been used to mitigate RAGE signaling. While others have proposed the use of oHSV and RAGE-targeting therapies such as anti-RAGE peptides[40] and soluble RAGE[41,42] as individual cancer treatment modalities, the combination into a single therapeutic has

not been explored for GBM therapy. Our *in vivo* observations in GBM-bearing mice treated with OVesRAGE revealed a better anti-tumor response toward oHSV therapy, underscoring the significance of this signaling pathway in oHSV therapy. Future studies would tease out the functional relevance of the different signaling pathways downstream of extracellular HMGB1-RAGE that impact oHSV therapy.

MATERIALS AND METHODS

Ethics statement

All animal studies were approved by the Center for Laboratory Animal Medicine and Care (CLAMC) at The University of Texas Health Science Center at Houston (AWC-18-0059, June 26th, 2018).

Cell lines and oHSV-1

U251T3, LN229, U87ΔEGFR, and Vero cells were maintained in Dulbecco's modified Eagle's medium (DMEM; Gibco BRL, Grand Island, NY, USA) supplemented with 10% fetal bovine serum (FBS). LN229 and U251 cells were obtained from Dr. Erwin G. Van Meir (Emory University, Atlanta, GA, USA), and U251-T3 cells were created in our laboratory (May 2009) as a tumorigenic clone of U251 cells by serially passaging these cells three times in mice. LN229 and T98G cells were obtained in January 2005 from Erwin G. Van Meir (Emory University). The U87ΔEGFR cell line expresses a truncated, constitutively active, mutant form of epidermal growth factor receptor (EGFRvIII) and has been previously described.[43] Monkey kidney epithelial-derived Vero cells have not been authenticated since receipt. Patient-derived primary GBM cells (GBM6, GBM8, GBM12, GBM28, GBM39, GBM59) were kindly provided by Dr. Jann N. Sarkaria (Mayo Clinic, Rochester, MN, USA) and were maintained as tumor spheres in neurobasal medium supplemented with 2% B27 without vitamin A, human EGF (20 ng/mL), and basic FGF (20 ng/mL) in low-attachment cell culture flasks. Monkey kidney epithelial-derived Vero cells were obtained from the ATCC. Primary HUVECs were purchased from ScienCell and cultured in EC medium (ECM; ScienCell, San Diego, CA, USA) as previously described.[27,44] Primary GBM8, GBM12 (October 2018), GBM6, GBM28, GBM39, and GBM59 cells (December 2021) were authenticated by the Cytogenetics and Cell Authentication Core at MD Anderson Cancer Center via short tandem repeat (STR) profiling. LN229 and U87ΔEGFR (October 2018) and U251T3 (January 2015) cells were authenticated by the University of Arizona Genetics Core. All cells are routinely monitored for changes in morphology and growth rate. All cells were maintained below passage 40 and are negative for mycoplasma. We used previously described technology[45] to generate OVesRAGE,[46] All viruses were propagated in Vero cells, and their respective titer (PFU/mL) was quantified using the PFU assay in Vero cells as previously described.[46]

Co-culture assay for virus replication assay, ELISA, and quantitative real-time PCR

Glioma cell lines and/or primary GBM cells were plated in 6-well plates. On the next day, cells were infected with virus at an MOI[47] of 0.1–0.5. One h after infection, unbound virus was removed and washed with PBS, and then cells were overlaid with serum-starved HUVECs (1:1 ratio of HUVECs to GBM cells). For virus replication assay, 48 h after co-culture, cells and media were collected, frozen, and thawed three times to release the viruses. The number of infectious particles present in the resulting supernatant was determined by performing a standard plaque-formation assay on Vero cells as described.[27,44] For ELISA and quantitative real-time PCR, 24 h after co-culture, cells and media were harvested and centrifuged for 5 min at 8,000 RPM, and cell pellets and supernatant CMs were frozen. Cells were used for quantitative real-time PCR, and CMs were used for esRAGE ELISA (cat. #K1009-1, B-Bridge International, Santa Clara, CA, USA). All assays were performed in triplicate.

Real-time PCR

Cell pellets were homogenized using a QIAshredder (Qiagen, Valencia, CA, USA), and RNA was isolated using RNeasy Mini Kit (Qiagen). Real-time continuous detection of PCR product was achieved using SYBR Green (Applied Biosystems, Carlsbad, CA, USA). GAPDH was used as an internal control with relative quantification being expressed as a ratio of the difference in the number of cycles needed for expression of a gene. Primers were designed using the Primer Express Program (Applied Biosystems) (Table S1).

EC migration assay

U251T3 cells were infected with the indicated virus at an MOI of 1. One h post infection, unbound virus was washed away, and serum-free media were added. Fourteen h post virus infection, CM was collected, treated with 0.4% human IgG to neutralize contaminated oHSV, and centrifuged for 10 min at 13,000 RPM to pellet any virus in the media. EC migration assays were performed using a modified Boyden chamber (8-μm pore size) from Corning Costar (Cambridge, MA, USA) similar to previous reports.[27,44] Migration of serum-starved HUVECs toward CM was measured using Transwell chambers. HUVECs were plated in the upper chamber, and cells were allowed to migrate for 6 h, at which point membranes were fixed in 1% glutaraldehyde and stained with 0.5% crystal violet; unmigrated cells were removed from the top chamber using a cotton swab. Images of the membranes were obtained at 20× magnification and quantified by counting 3 fields of view per well (n = 3/group).

EC adhesion assay

CM was collected as described in the above EC migration assay. Adhesion of a human donor PBMC to an HUVEC was conducted using CytoSelect leukocyte-endothelium adhesion assay kit (Cell Biolabs, San Diego, CA, USA) per the manufacturer's instructions. Briefly, PBMC adhesion to the HUVEC monolayer was conducted using CytoSelect Leukocyte-Endothelium Adhesion Assay kit. Glioma cells treated with PBS or rHSVQ for 1 h were incubated with isotype or 50 μg/mL HMGB1 blocking or isotype control chicken IgY antibodies (IBL International, Toronto, ON, Canada), 2 μg/mL RAGE antibody (cat. #sc-365154) or isotype control mouse IgG antibodies (Santa Cruz Biotechnology, Dallas, TX, USA), or 200 ng/mL esRAGE (BioVendor, cat. #RD172116100-HEK, Brno, Czech Republic) for 6 h before harvesting CM. HUVECs grown for 72 h were washed three times with HBSS and treated with the harvested CM.

LeukoTracker-stained PBMCs obtained from donor human blood were layered onto stimulated HUVECs. PBMCs were allowed to adhere at 37°C for 1 h, and unbound cells were washed away. Fluorescence of lysed labeled PBMCs was measured using microplate reader. Experiment was repeated twice in triplicate.

Western blot analysis and antibodies

Cell lysates were fractionated by SDS-PAGE and transferred to nitrocellulose (NC) membranes. Blocked membranes were then incubated with antibodies against anti-RAGE (H-300) (cat. #sc-5563) from Santa Cruz Biotechnology (Santa Cruz, CA, USA) and anti-cleaved PARP (cat. #9541), anti-phosphor MEK1/2 (cat. #9154), anti-phosphor Erk (cat. #9271), anti-total Erk (cat. #4370), anti-total MEK1/2 (cat. #9126), anti-GAPDH (Cat. #2118), and HRP-conjugated secondary anti-rabbit antibody (cat. #7074S) from Cell Signaling Technology (Danvers, MA, USA), and the immunoreactive bands were visualized using enhanced chemiluminescence (ECL) (GE Healthcare, Piscataway, NJ, USA). Original full-length images of western blotting analysis are provided (Figures S1–S3). Details of the antibodies used in the study are listed in Table S2.

Flow cytometry

ECs and HUVECs were plated on 6 well plates. Six h later, stably mCherry-expressing U251T3 cells (U251T3-mCherry) were collected and infected with 0.1 MOI rHSVQ or OVesRAGE virus for 30 min in a suspension condition. Cells were washed and overlaid on top of an equivalent number of ECs and cultured for 24 h. Cells were then collected and stained with Live/Dead Fixable Aqua Dead Cell Stain Kit (Life Technologies, Eugene, OR, USA) and analyzed in CytoFlex (Beckman Coulter, Brea, CA, USA). Single-stain controls for each fluorochrome were prepared using cells or compensation beads (Invitrogen, cat #01-222-42, Waltham, MA, USA) for compensation. Tumor and ECs were initially separated by gating for the mCherry-positive population (550–650 nm emission) and then further gated for GFP expression and live/dead cell staining-positive population. GFP expression represents virus-infected cells, while live/dead staining represents dead cell population. Data were analyzed using FlowJo v.10.7.

Animal surgery

All mice were housed and experiments were performed in accordance with the Subcommittee on Research Animal Care of the Ohio State University[48] guidelines and the Animal Welfare Committee at the University of Texas Health Science Center in Houston guidelines and have been approved by the Institutional Review Board. Six-week-old outbred female athymic nu/nu mice were obtained from the Target Validation Shared Resource at the Ohio State University, from which original breeders (strains #553 and #554) were received from the NCI Frederick facility. NSG mice were obtained from Jackson Laboratory. Anesthetized mice were fixed in a stereotactic apparatus, and a burr hole was drilled at 2-mm lateral and 1-mm front from the bregma to a depth of 3.5 mm. 1×10^5 U87ΔEGFR cells or GBM30 in 2 μL PBS were implanted. Seven days post tumor cell implantation, mice were randomized, anesthetized again, and injected intratumorally with PBS or 2×10^5 PFU rHSVQ or OVesRAGE at the same location. Animals were observed daily and euthanized at the indicated time points or when they showed a body score of 2 or less.

Statistical analysis

To compare two independent treatments for continuous endpoints such as viral titers, cell viability assay, Cytation, ELISA, and quantitative real-time PCR, Student's t test was used. When multiple pairwise comparisons are made, one-way ANOVA was used. Log rank test was used to compare survival curves for survival data. p values were adjusted for multiple comparisons by Holms' procedure. A p value of 0.05 or less is considered significant.

DATA AVAILABILITY

All data herein described are included in this published article.

SUPPLEMENTAL INFORMATION

Supplemental information can be found online at https://doi.org/10.1016/j.omto.2023.01.003.

ACKNOWLEDGMENTS

This work was supported by Research Scholar Grants (RSG-19-185-01-MPC) from the American Cancer Society and R21 NS123685-01A1 from the National Institutes of Health (NIH) to J.Y.Y.; by R01 CA150153, R61NS112410, and P01CA163205-08 from the NIH as well as W81XWH-21-10068 from the Department of Defense to B.K.; by a High Impact/High Risk grant (RP200615) from the Cancer Prevention and Research Institute of Texas (CPRIT) and R03 CA252770 from NIH to T.J.L.; and by the American Cancer Society/Joe & Jessie Crump Foundation and North Texas Pay-if Group to J.S. K.A.R.-C. is a CPRIT Predoctoral Fellow in the Biomedical Informatics, Genomics and Translational Cancer Research Training Program (BIG-TCR) funded by the CPRIT (CPRIT RP210045).

AUTHOR CONTRIBUTIONS

J.Y.Y. and B.K. conceptualized and designed the studies. J.S., J.S.S., K.A.R.-C., K.V.-A., B.H., A.J.B.-P., T.J.L, Y.K.B.-S., and J.Y.Y. performed the experiments. J.S., J.S.S., K.A.R.-C., K.V.-A., B.H., A.J.B.-P., T.J.L., Y.K.B.-S., and J.Y.Y. analyzed data. B.K. and J.Y.Y. wrote the manuscript. All authors edited and reviewed the manuscript.

DECLARATION OF INTERESTS

The authors declare no competing interests.

REFERENCES

1. Delgado-López, P.D., and Corrales-García, E.M. (2016). Survival in glioblastoma: a review on the impact of treatment modalities. Clin. Transl. Oncol. *18*, 1062–1071.

2. Grech, N., Dalli, T., Mizzi, S., Meilak, L., Calleja, N., and Zrinzo, A. (2020). Rising incidence of glioblastoma multiforme in a well-defined population. Cureus *12*, e8195.

3. Stylli, S.S., Luwor, R.B., Ware, T.M.B., Tan, F., and Kaye, A.H. (2015). Mouse models of glioma. J. Clin. Neurosci. *22*, 619–626.

4. Andtbacka, R.H.I., Kaufman, H.L., Collichio, F., Amatruda, T., Senzer, N., Chesney, J., Delman, K.A., Spitler, L.E., Puzanov, I., Agarwala, S.S., et al. (2015). Talimogene laherparepvec improves durable response rate in patients with advanced melanoma. J. Clin. Oncol. *33*, 2780–2788.

5. Killock, D. (2015). Skin cancer: T-VEC oncolytic viral therapy shows promise in melanoma. Nat. Rev. Clin. Oncol. *12*, 438.

6. Zheng, M., Huang, J., Tong, A., and Yang, H. (2019). Oncolytic viruses for cancer therapy: barriers and recent advances. Mol. Ther. Oncolytics *15*, 234–247.

7. Hong, B., Muili, K., Bolyard, C., Russell, L., Lee, T.J., Banasavadi-Siddegowda, Y., Yoo, J.Y., Yan, Y., Ballester, L.Y., Bockhorst, K.H., and Kaur, B. (2019). Suppression of HMGB1 released in the glioblastoma tumor microenvironment reduces tumoral edema. Mol. Ther. Oncolytics *12*, 93–102.

8. Sprague, L., Lee, J.M., Hutzen, B.J., Wang, P.Y., Chen, C.Y., Conner, J., Braidwood, L., Cassady, K.A., and Cripe, T.P. (2018). High mobility group Box 1 influences HSV1716 spread and acts as an adjuvant to chemotherapy. Viruses *10*, 132.

9. Pan, C., Cai, Q., Li, X., Li, L., Yang, L., Chen, Y., Liu, J., Liu, W., Gao, M., Sui, T., et al. (2022). Correction to: enhancing the HSV-1-mediated antitumor immune response by suppressing Bach1. Cell. Mol. Immunol. *19*, 754.

10. Thomas, S., Kuncheria, L., Roulstone, V., Kyula, J.N., Mansfield, D., Bommareddy, P.K., Smith, H., Kaufman, H.L., Harrington, K.J., and Coffin, R.S. (2019). Development of a new fusion-enhanced oncolytic immunotherapy platform based on herpes simplex virus type 1. J. Immunother. Cancer *7*, 214.

11. Bommareddy, P.K., Zloza, A., Rabkin, S.D., and Kaufman, H.L. (2019). Oncolytic virus immunotherapy induces immunogenic cell death and overcomes STING deficiency in melanoma. Oncoimmunology *8*, 1591875.

12. Takasu, A., Masui, A., Hamada, M., Imai, T., Iwai, S., and Yura, Y. (2016). Immunogenic cell death by oncolytic herpes simplex virus type 1 in squamous cell carcinoma cells. Cancer Gene Ther. *23*, 107–113.

13. Workenhe, S.T., Simmons, G., Pol, J.G., Lichty, B.D., Halford, W.P., and Mossman, K.L. (2014). Immunogenic HSV-mediated oncolysis shapes the antitumor immune response and contributes to therapeutic efficacy. Mol. Ther. *22*, 123–131.

14. Sparvero, L.J., Asafu-Adjei, D., Kang, R., Tang, D., Amin, N., Im, J., Rutledge, R., Lin, B., Amoscato, A.A., Zeh, H.J., and Lotze, M.T. (2009). RAGE (Receptor for Advanced Glycation Endproducts), RAGE ligands, and their role in cancer and inflammation. J. Transl. Med. *7*, 17.

15. Hoesel, B., and Schmid, J.A. (2013). The complexity of NF-kappaB signaling in inflammation and cancer. Mol. Cancer *12*, 86.

16. Tesarová, P., Kalousová, M., Jáchymová, M., Mestek, O., Petruzelka, L., and Zima, T. (2007). Receptor for advanced glycation end products (RAGE)–soluble form (sRAGE) and gene polymorphisms in patients with breast cancer. Cancer Invest. *25*, 720–725.

17. Gu, H., Yang, L., Sun, Q., Zhou, B., Tang, N., Cong, R., Zeng, Y., and Wang, B. (2008). Gly82Ser polymorphism of the receptor for advanced glycation end products is associated with an increased risk of gastric cancer in a Chinese population. Clin. Cancer Res. *14*, 3627–3632.

18. Chen, X., Zhang, L., Zhang, I.Y., Liang, J., Wang, H., Ouyang, M., Wu, S., da Fonseca, A.C.C., Weng, L., Yamamoto, Y., et al. (2014). RAGE expression in tumor-associated macrophages promotes angiogenesis in glioma. Cancer Res. *74*, 7285–7297.

19. Wagner, N.B., Weide, B., Reith, M., Tarnanidis, K., Kehrel, C., Lichtenberger, R., Pflugfelder, A., Herpel, E., Eubel, J., Ikenberg, K., et al. (2015). Diminished levels of the soluble form of RAGE are related to poor survival in malignant melanoma. Int. J. Cancer *137*, 2607–2617.

20. Yamamoto, M., Yamaguchi, T., Yamauchi, M., and Sugimoto, T. (2009). Low serum level of the endogenous secretory receptor for advanced glycation end products (esRAGE) is a risk factor for prevalent vertebral fractures independent of bone mineral density in patients with type 2 diabetes. Diabetes Care *32*, 2263–2268.

21. Otani, Y., Yoo, J.Y., Chao, S., Liu, J., Jaime-Ramirez, A.C., Lee, T.J., Hurwitz, B., Yan, Y., Dai, H., Glorioso, J.C., et al. (2020). Oncolytic HSV-infected glioma cells activate NOTCH in adjacent tumor cells sensitizing tumors to gamma secretase inhibition. Clin. Cancer Res. *26*, 2381–2392.

22. Arefian, E., Bamdad, T., Soleimanjahi, H., Sabahi, F., Akhoond, M.R., Parsania, M., and Ghaemi, A. (2007). Evaluation of gamma-interferon kinetics in HSV-1 infected mice in different days post infection (in vivo) and post re-stimulation (in vitro). Comp. Immunol. Microbiol. Infect. Dis. *30*, 1–9.

23. Bowman, R.L., Wang, Q., Carro, A., Verhaak, R.G.W., and Squatrito, M. (2017). GlioVis data portal for visualization and analysis of brain tumor expression datasets. Neuro. Oncol. *19*, 139–141.

24. Russell, L., Swanner, J., Jaime-Ramirez, A.C., Wang, Y., Sprague, A., Banasavadi-Siddegowda, Y., Yoo, J.Y., Sizemore, G.M., Kladney, R., Zhang, J., et al. (2018). PTEN expression by an oncolytic herpesvirus directs T-cell mediated tumor clearance. Nat. Commun. *9*, 5006.

25. da Fonseca, A.C.C., and Badie, B. (2013). Microglia and macrophages in malignant gliomas: recent discoveries and implications for promising therapies. Clin. Dev. Immunol. *2013*, 264124.

26. Conry, R.M., Westbrook, B., McKee, S., and Norwood, T.G. (2018). Talimogene laherparepvec: first in class oncolytic virotherapy. Hum. Vaccin. Immunother. *14*, 839–846.

27. Nair, M., Khosla, M., Otani, Y., Yeh, M., Park, F., Shimizu, T., Kang, J.M., Bolyard, C., Yu, J.G., Kumar Banasavadi-Siddegowda, Y., et al. (2020). Enhancing antitumor efficacy of heavily vascularized tumors by RAMBO virus through decreased tumor endothelial cell activation. Cancers (Basel) *12*, 1040.

28. Alvarez-Breckenridge, C., Kaur, B., and Chiocca, E.A. (2009). Pharmacologic and chemical adjuvants in tumor virotherapy. Chem. Rev. *109*, 3125–3140.

29. Shayan, S., Arashkia, A., Bahramali, G., Abdoli, A., Nosrati, M.S.S., and Azadmanesh, K. (2022). Cell type-specific response of colon cancer tumor cell lines to oncolytic HSV-1 virotherapy in hypoxia. Cancer Cell Int. *22*, 164.

30. Gatson, N.N., Chiocca, E.A., and Kaur, B. (2012). Anti-angiogenic gene therapy in the treatment of malignant gliomas. Neurosci. Lett. *527*, 62–70.

31. Wang, H., Zhang, L., Zhang, I.Y., Chen, X., Da Fonseca, A., Wu, S., Ren, H., Badie, S., Sadeghi, S., Ouyang, M., et al. (2013). S100B promotes glioma growth through chemoattraction of myeloid-derived macrophages. Clin. Cancer Res. *19*, 3764–3775.

32. Carvalho da Fonseca, A.C., Wang, H., Fan, H., Chen, X., Zhang, I., Zhang, L., Lima, F.R.S., and Badie, B. (2014). Increased expression of stress inducible protein 1 in glioma-associated microglia/macrophages. J. Neuroimmunol. *274*, 71–77.

33. Kierdorf, K., and Fritz, G. (2013). RAGE regulation and signaling in inflammation and beyond. J. Leukoc. Biol. *94*, 55–68.

34. Jeong, J., Lee, J., Lim, J., Cho, S., An, S., Lee, M., Yoon, N., Seo, M., Lim, S., and Park, S. (2019). Soluble RAGE attenuates AngII-induced endothelial hyperpermeability by disrupting HMGB1-mediated crosstalk between AT1R and RAGE. Exp. Mol. Med. *51*, 1–15.

35. Webster, L., Abordo, E.A., Thornalley, P.J., and Limb, G.A. (1997). Induction of TNF alpha and IL-1 beta mRNA in monocytes by methylglyoxal- and advanced glycated endproduct-modified human serum albumin. Biochem. Soc. Trans. *25*, 250S.

36. Taguchi, A., Blood, D.C., del Toro, G., Canet, A., Lee, D.C., Qu, W., Tanji, N., Lu, Y., Lalla, E., Fu, C., et al. (2000). Blockade of RAGE-amphoterin signalling suppresses tumour growth and metastases. Nature *405*, 354–360.

37. Haseley, A., Boone, S., Wojton, J., Yu, L., Yoo, J.Y., Yu, J., Kurozumi, K., Glorioso, J.C., Caligiuri, M.A., and Kaur, B. (2012). Extracellular matrix protein CCN1 limits oncolytic efficacy in glioma. Cancer Res. *72*, 1353–1362.

38. Thorne, A.H., Meisen, W.H., Russell, L., Yoo, J.Y., Bolyard, C.M., Lathia, J.D., Rich, J., Puduvalli, V.K., Mao, H., Yu, J., et al. (2014). Role of cysteine-rich 61 protein (CCN1) in macrophage-mediated oncolytic herpes simplex virus clearance. Mol. Ther. *22*, 1678–1687.

39. Bolyard, C., Meisen, W.H., Banasavadi-Siddegowda, Y., Hardcastle, J., Yoo, J.Y., Wohleb, E.S., Wojton, J., Yu, J.G., Dubin, S., Khosla, M., et al. (2017). BAI1 orchestrates macrophage inflammatory response to HSV infection-implications for oncolytic viral therapy. Clin. Cancer Res. *23*, 1809–1819.

40. Arumugam, T., Ramachandran, V., Gomez, S.B., Schmidt, A.M., and Logsdon, C.D. (2012). S100P-derived RAGE antagonistic peptide reduces tumor growth and metastasis. Clin. Cancer Res. *18*, 4356–4364.

41. Schmidt, A.M. (2015). Soluble RAGEs - prospects for treating & tracking metabolic and inflammatory disease. Vascul. Pharmacol. *72*, 1–8.

42. DiNorcia, J., Moroziewicz, D.N., Ippagunta, N., Lee, M.K., Foster, M., Rotterdam, H.Z., Bao, F., Zhou, Y.S., Yan, S.F., Emond, J., et al. (2010). RAGE signaling

significantly impacts tumorigenesis and hepatic tumor growth in murine models of colorectal carcinoma. J. Gastrointest. Surg. *14*, 1680–1690.

43. Narita, Y., Nagane, M., Mishima, K., Huang, H.J.S., Furnari, F.B., and Cavenee, W.K. (2002). Mutant epidermal growth factor receptor signaling down-regulates p27 through activation of the phosphatidylinositol 3-kinase/Akt pathway in glioblastomas. Cancer Res. *62*, 6764–6769.

44. Nair, M., Bolyard, C., Lee, T.J., Kaur, B., and Yoo, J.Y. (2021). Therapeutic application of brain-specific angiogenesis inhibitor 1 for cancer therapy. Cancers (Basel) *13*, 3562.

45. Terada, K., Wakimoto, H., Tyminski, E., Chiocca, E.A., and Saeki, Y. (2006). Development of a rapid method to generate multiple oncolytic HSV vectors and their in vivo evaluation using syngeneic mouse tumor models. Gene Ther. *13*, 705–714.

46. Wakimoto, H., Fulci, G., Tyminski, E., and Chiocca, E.A. (2004). Altered expression of antiviral cytokine mRNAs associated with cyclophosphamide's enhancement of viral oncolysis. Gene Ther. *11*, 214–223.

47. Ferguson, M.S., Lemoine, N.R., and Wang, Y. (2012). Systemic delivery of oncolytic viruses: hopes and hurdles. Adv. Virol. *2012*, 805629.

48. Koga, K., Izumi, G., Mor, G., Fujii, T., and Osuga, Y. (2014). Toll-like receptors at the maternal-fetal interface in normal pregnancy and pregnancy complications. Am. J. Reprod. Immunol. *72*, 192–205.

OMTO, Volume *28*

Supplemental information

esRAGE-expressing oHSV enhances anti-tumor efficacy by inhibition of endothelial cell activation

Jessica Swanner, Ji Seon Shim, Kimberly A. Rivera-Caraballo, Karina Vázquez-Arreguín, Bangxing Hong, Alberto J. Bueso-Perez, Tae Jin Lee, Yeshavanth Kumar Banasavadi-Siddegowda, Balveen Kaur, and Ji Young Yoo

Supplemental Materials

Figure S1.

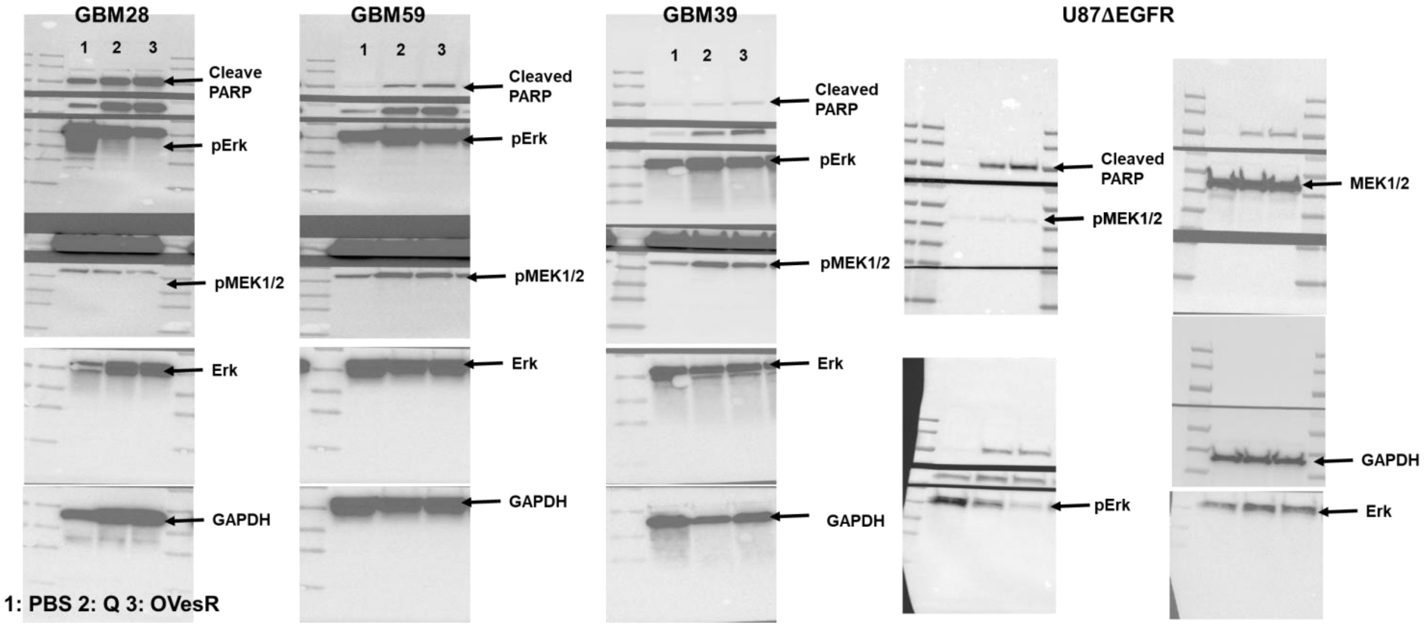

Figure S1. Original full-length image of western blotting analysis used in the Figure 4E. The immunoreactive bands on nitrocellulose membranes were visualized with enhanced chemiluminescence (ECL) (GE Healthcare, Piscataway, NJ, USA) using ChemiDoc MP (Bio-Rad, Hercules, CA, USA). Lane 1: PBS; Lane 2: Q; Lane 3: OVesR.

Figure S2.

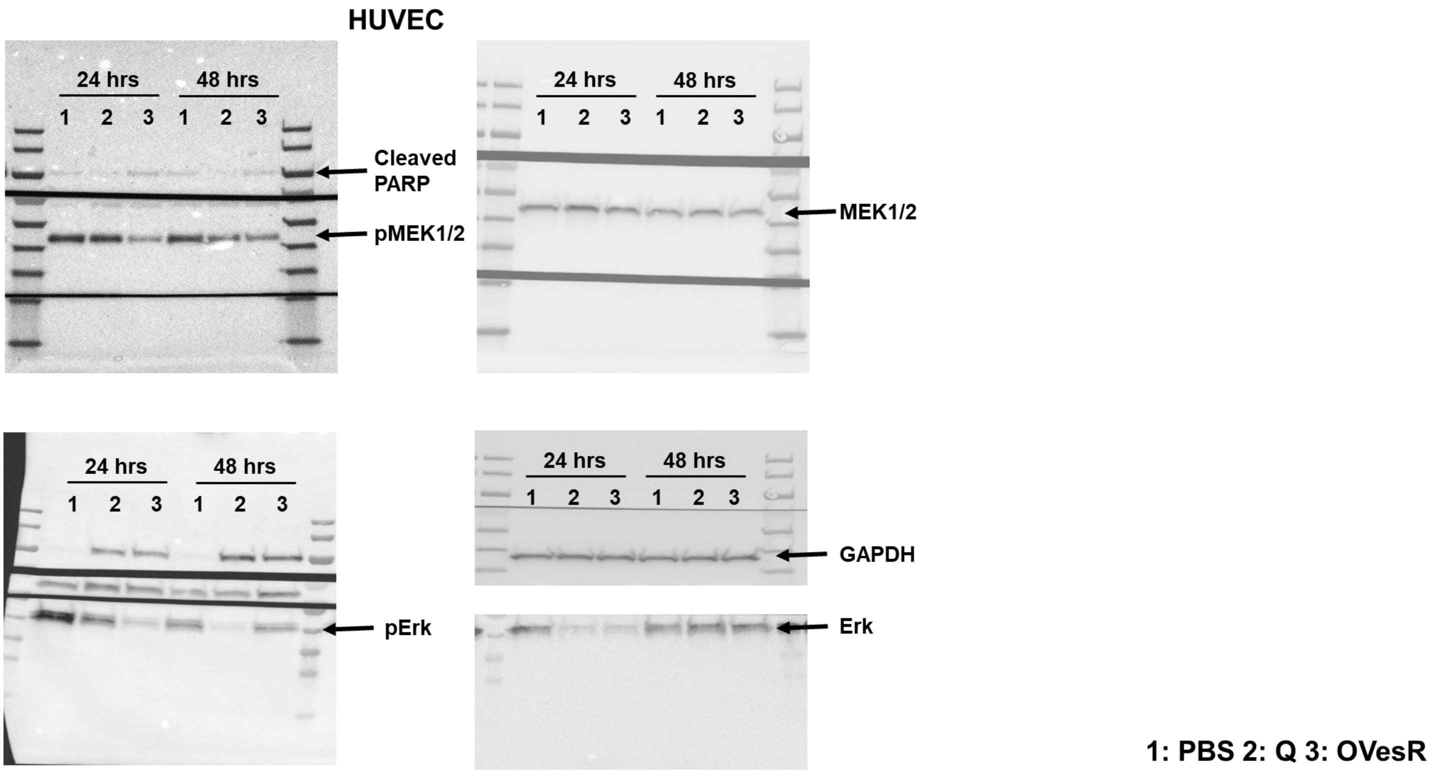

Figure S2. Original full-length image of western blotting analysis used in the Figure 5C. The immunoreactive bands on nitrocellulose membranes were visualized with enhanced chemiluminescence (ECL) (GE Healthcare, Piscataway, NJ, USA) using ChemiDoc MP (Bio-Rad, Hercules, CA, USA). Lane 1: PBS; Lane 2: Q; Lane 3: OVesR.

Figure S3.

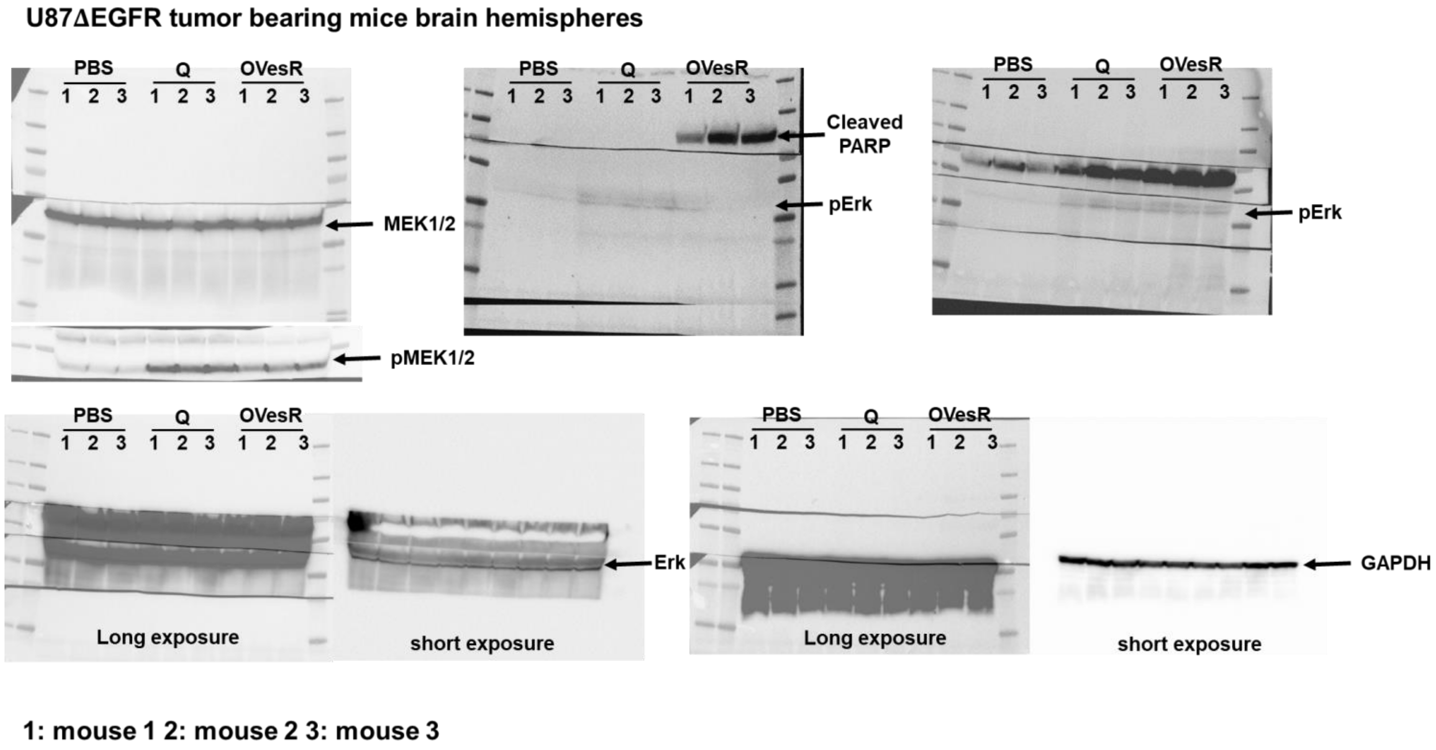

Figure S3. Original full-length image of western blotting analysis used in the Figure 6B. The immunoreactive bands on nitrocellulose membranes were visualized with enhanced chemiluminescence (ECL) (GE Healthcare, Piscataway, NJ, USA) using ChemiDoc MP (Bio-Rad, Hercules, CA, USA). Long and short exposure images were provided for total Erk and GAPDH. Numbers 1, 2, and 3 stand for each mouse.

Table S1. List of the primers used in the study

hCCL5	Forward	5'-CCTGCTGCTTTGCCTACATTGC-3'
	Reverse	5'-ACACACTTGGCGGTTCTTTCGG-3'
hVCAM1	Forward	5'-GTGGAGCTCTACTCATTCCCTA-3'
	Reverse	5'-CTCCAGAATAGTCTCCCCCTTAAG-3'
hICAM1	Forward	5'-GGTAGCAGCCGCAGTCATAA-3'
	Reverse	5'-TCCCTTTTTGGGCCTGTTGT-3'
GAPDH	Forward	5'-CCAGCAAGGACACTGAGCAA-3'
	Reverse	5'-GGGATGGAAATTGTGAGGGA-3'

Table S2. List of the antibodies used in the study

Antibody	Cat#	Vendor	Dilution
Cleaved PARP	9541	Cell signaling Technology	1:1000
p-AKT	9271	Cell signaling Technology	1:1000
p-ERK	4370	Cell signaling Technology	1:1000
T-ERK	9102	Cell signaling Technology	1:1000
T-AKT	4691	Cell signaling Technology	1:1000
p-MEK	9154	Cell signaling Technology	1:1000
GAPDH	2118	Cell signaling Technology	1:2000
T-MEK	9126	Cell signaling Technology	1:1000
Anti-rabbit IgG, HRP-linked Antibody	7074	Cell signaling Technology	1:2000
